# Exposure to environmentally persistent free radicals during gestation lowers energy expenditure and impairs skeletal muscle mitochondrial function in adult mice

**DOI:** 10.1152/ajpendo.00521.2015

**Published:** 2016-04-26

**Authors:** Erin J. Stephenson, Alyse Ragauskas, Sridhar Jaligama, JeAnna R. Redd, Jyothi Parvathareddy, Matthew J. Peloquin, Jordy Saravia, Joan C. Han, Stephania A. Cormier, Dave Bridges

**Affiliations:** ^1^Department of Physiology, University of Tennessee Health Science Center, Memphis, Tennessee;; ^2^Department of Pediatrics, University of Tennessee Health Science Center, Memphis, Tennessee; and; ^3^Children's Foundation Research Institute, Le Bonheur Children's Hospital, Memphis, Tennessee

**Keywords:** in utero exposure, environmentally persistent free radicals, oxidative stress, skeletal muscle, mitochondria

## Abstract

We have investigated the effects of in utero exposure to environmentally persistent free radicals (EPFRs) on growth, metabolism, energy utilization, and skeletal muscle mitochondria in a mouse model of diet-induced obesity. Pregnant mice were treated with laboratory-generated, combustion-derived particular matter (MCP230). The adult offspring were placed on a high-fat diet for 12 wk, after which we observed a 9.8% increase in their body weight. The increase in body size observed in the MCP230-exposed mice was not associated with increases in food intake but was associated with a reduction in physical activity and lower energy expenditure. The reduced energy expenditure in mice indirectly exposed to MCP230 was associated with reductions in skeletal muscle mitochondrial DNA copy number, lower mRNA levels of electron transport genes, and reduced citrate synthase activity. Upregulation of key genes involved in ameliorating oxidative stress was also observed in the muscle of MCP230-exposed mice. These findings suggest that gestational exposure to MCP230 leads to a reduction in energy expenditure at least in part through alterations to mitochondrial metabolism in the skeletal muscle.

obesity is a major global health concern, and emerging data support a role for environmental pollutants in the pathogenesis of obesity and its comorbidities (2, 7, 9, 10, 12, 22, 24, 45). Gestational and early-life exposure to combustion-derived particulate matter (PM) has been associated with an increased risk of obesity in humans (9, 12, 18, 22, 24). This association is supported by data obtained from animal studies, where the offspring of pregnant mice, which have been exposed to diesel exhaust in utero, are predisposed to weight gain as adults (6). Furthermore, several studies have linked the exposure to combustion-derived PM to impaired metabolic health in humans (2, 7, 10, 45) and animals (29, 30, 43, 55, 56). Specifically, cross-sectional studies of human subjects that are chronically exposed to combustion-derived PM have shown associations with type 2 diabetes and cardiovascular disease (2, 7, 45), whereas murine models of chronic PM exposure indicate that pollutants lead to elevated adipose tissue inflammation and insulin resistance (30, 43, 55). Relatively stable radicals with half-lives of ∼21 days exist on the surface of airborne PM (13, 15, 31), and these are referred to as environmentally persistent free radicals (EPFRs).

From a mechanistic stand point, exactly how environmental pollutants result in obesity and other metabolic abnormalities is currently unknown. However, mitochondrial deficiencies and structural abnormalities have been observed in adipose tissue (55, 56), vascular tissue (53), and cardiac muscle (28) following exposure to combustion-derived pollutants that should contain EPFRs. Mitochondria are responsible for oxidative cellular energy production and endogenous reactive oxygen species production and are an essential component of the antioxidant defense system (23). Thus, defects in mitochondrial metabolism, particularly in the context of obesity, are likely to have profound effects on energy homeostasis and other metabolic pathways. The importance of skeletal muscle mitochondrial metabolism for maintaining metabolic health is becoming well recognized (21, 37, 41) with deficits in muscle quality and function, particularly during early development (8), being closely linked to later life metabolic disturbances such as impaired growth or insulin resistance (16, 37). However, the effects of in utero exposure to EPFRs on skeletal muscle mitochondrial quality remain to be determined. In this study, we investigated the effects of in utero exposure to EPFRs on growth, metabolism, energy utilization, and skeletal muscle mitochondria in a mouse model of diet-induced obesity. We hypothesized that gestational exposure to EPFRs reduces energy expenditure and results in mitochondrial impairments in the skeletal muscle.

## MATERIALS AND METHODS

### 

#### MCP230 preparation.

EPFR-containing particles [i.e., laboratory-generated, combustion-derived particular matter (MCP230)] were generated and characterized by our colleagues, as described previously (31). Suspensions of MCP230 and cabosil, a non EPFR-containing amorphous silica particle control (1 mg/ml), were prepared in irrigation saline containing 0.02% Tween 80, and the resulting particle suspension was monodispersed by probe sonication.

#### Animals, particulate exposure, and high-fat diet.

C57BL/6NHsd mice were purchased from Harlan (Indianapolis, IN). Mice were maintained in a 12-h light-dark cycle room at constant temperature and humidity and allowed unrestricted access to food and water. Breeder mice (6 wk of age) were mated and pregnant dams administered 50 μl of MCP230 particle suspension via oropharyngeal aspiration on *days 10* and *17* of gestation, as described earlier (51). Control mice received 50 μl of saline or cabosil. Pregnant mice were anesthetized by inhalant anesthetic isoflurane (5%) and placed in a holder and physically supported in an upright position. The suspension was instilled just above the vocal cords while the tongue was held with forceps to prevent swallowing. Offspring were weaned at 4 wk of age. Male mice were selected for the study and were fed standard rodent chow until 10 wk of age. At 10 wk of age, mice were switched from chow to a high-fat diet (HFD) consisting of 45% of calories from fat (catalog D12451; Research Diets). Mice were maintained on HFD for 12 wk. One mouse, an MCP230-treated animal, had malocclusion and was removed from all data analyses. The University of Tennessee Health Science Center Institutional Animal Care and Use Committee approved all mouse procedures.

#### Metabolite assays.

Blood was collected in both the fed and 16-h-fasted state. Blood glucose was determined using an AccuCheck glucometer. Serum hormone levels were determined using a Bio-Plex pro mouse diabetes multiplex immunoassay (no. 171-F7001M; Bio-Rad), following the manufacturer's instructions using a MAGPIX LMX200 system. Homeostatic model assessment of insulin resistance (HOMA-IR) was calculated from 16-h-fasting glucose and insulin values.

#### Body composition and metabolic cages.

Mice were weighed weekly from approximately Zeitgerber time (ZT)10. Body composition was determined noninvasively using an echo-MRI 100. Food intake during the HFD phase was determined on a per-cage level by weighing the food on a weekly basis. For food intake pre-HFD, this was determined by scaled feeders within the Comprehensive Laboratory Animal Monitoring System (CLAMS) system.

V̇o_2_, energy expenditure, ambulatory locomotor activity, and respiratory exchange ratios were determined in a home cage style CLAMS (Columbus Instruments). Mice were placed in the cages at approximately ZT10 and monitored for 3–4 days. Data from the first 6 h were discarded, as this was the amount of time determined to be necessary for the mice to acclimate to their new environment. Oxymax software (Columbus Instruments) calculated the volumes of O_2_, CO_2_, the respiratory exchange ratio, the ambulatory x- and y-phase physical activity, and the food consumption. Heat production was calculated using the Lusk equation (32) via the Oxymax software: heat = (3.815 + 1.232 × RER) × V̇o_2_.

#### Tissue collection and nucleic acid preparation.

After the 12-wk HFD phase, mice were fasted overnight and anesthetized with ketamine-xylazine (180:10 mg/kg, respectively), which was delivered intraperitoneally. Quadriceps muscles were carefully dissected out, cleared of any visible adipose and connective tissue, and snap-frozen in liquid N_2_. Nucleic acids were isolated from frozen quadriceps samples via Trizol extraction using a Qiagen Tissue Lyser (30 Hz for 5 min). Following careful and complete removal of the RNA-containing aqueous phase and its subsequent column purification (PureLink mRNA kit from Life Technologies), DNA extraction buffer [Tris base (1 M), sodium citrate dibasic trihydrate (50 mM), and guanidine thiocyanate (4 M)] was added to the tubes containing the remaining Trizol-separated interphase and infranatant. Tubes were shaken vigorously and centrifuged at 12,000 *g* at room temperature for 30 min. The aqueous phase was collected, and the genomic and mitochondrial DNA were precipitated in isopropanol. Samples were respun at 12,000 *g* at 4°C to pellet the DNA. The DNA pellet was then washed in 70% ethanol, respun, and, after careful ethanol removal, resuspended in TE buffer. cDNA was generated from purified RNA using the Applied Biosystems cDNA Synthesis Kit.

#### Quantitative PCR analysis of mitochondrial DNA copy number and mRNA transcripts.

Primers designed for three mitochondrial-encoded gene regions were used to assess mitochondrial DNA (mtDNA) copy number in DNA, and primers designed for quantitative RT-PCR were used to assess mRNA transcript levels (Table 1). Briefly, DNA or cDNA from each sample extraction was added to the appropriate working quantitative PCR master mix (containing SYBR Green and the relevant primers at a final concentration of 100 nM each). PCR conditions included an activation cycle of 95°C for 10 min followed by 45 amplification cycles of 15 s at 95°C, 15 s at 60°C, and 10 s at 73°C. C_p_ values were quantified on a Light Cycler 480. Nucleic acid levels were calculated using the ΔΔC_T_ method, with data for mtDNA copy number being normalized to values obtained for a nuclear-encoded genomic locus (*Tsc2*) and mRNA levels being normalized to *Rpl13a*, which was determined to be unaffected by MCP230 treatment compared with other commonly used normalization controls, including *Rplp0* and *Gapdh*.

**Table 1.** Mitochondrial DNA copy number and relative gene expression were determined using the following primer sequences

Region/gene	Forward Primer	Reverse Primer
d-Loop	GGC CCA TTA AAC TTG GGG GT	TTC TTC ACC GTA GGT GCG TC
*mt-Nd1*	CGT CCC CAT TCT AAT CGC CA	ATG GCG TCT GCA AAT GGT TG
*mt-Cytb*	CTT CAT GTC GGA CGA GGC TT	CCT CAT GGA AGG ACG TAG CC
*mt-Nd4*	TAA TCG CAC ATG GCC TCA CA	GCT GTG GAT CCG TTC GTA GT
*Sdha*	TCT TCG CTG GTG TGG ATG TC	CTT CAG CAC CTG TCC CTT GT
*mt-Co2*	AAC CGA GTC GTT CTG CCA AT	CTA GGG AGG GGA CTG CTC AT
*Ppard*	ACA TGG AAT GTC GGG TGT GC	CGG AAG AAG CCC TTG CAC C
*Ppargc1a*	TGA TGT GAA TGA CTT GGA TAC AGA CA	GCT CAT TGT TGT ACT GGT TGG ATA TG
*Ppargc1b*	TTG TAG AGT GCC AGG TGC TG	GTG TAT CTG GGC CAA CGG AA
*Nrf1*	AGA AAC GGA AAC GGC CTC AT	GGC TCT GAG TTT CCG AAG CA
*Nfe2l2*	TGG ACT TGG AGT TGC CAC C	TCT TGC CTC CAA AGG ATG TCA
*Tfam*	TCG CAT CCC CTC GTC TAT CA	AGT TTT GCA TCT GGG TGT TTA GC
*Ucp2*	TGC GGT CCG GAC ACA ATA G	GCC TCC AAG GTC AAG CTT CT
*Ucp3*	ACA AAG GAT TTG TGC CCT CC	TCA AAA CGG AGA TTC CCG CA
*Sod1*	GGA ACC ATC CAC TTC GAG CA	CCC ATG CTG GCC TTC AGT TA
*Sod2*	TTC TGG ACA AAC CTG AGC CC	GTC ACG CTT GAT AGC CTC CA
*Cat*	CAC TGA CGA GAT GGC ACA CT	TGT GGA GAA TCG AAC GGC AA
*Gpx1*	TTC GGA CAC CAG GAG AAT GG	TAA AGA GCG GGT GAG CCT TC
*Gclm*	TGG AGT TCC CAA ATC AGC CC	CAA CTC CAA GGA CGG AGC AT
*Tsc2*	AAG AAG CCT CTT CTG CTA CC	CAG CTC CGA CCA TGA AGT G
*Rpl13a*	GGA GTC CGT TGG TCT TGA GG	GGC CAA GAT GCA CTA TCG GA

*Tsc2* and *Rpl13a* were used for normalization of genomic DNA and mRNA, respectively.

#### Preparation of protein lysates and Western blotting.

Skeletal muscle homogenates were prepared from ∼30 to 50 mg of frozen quadriceps in RIPA buffer [Tris basic (50 mM), sodium deoxycholate (0.25%), NP-40 (1%), NaCl (150 mM), EDTA (1 mM), Na_3_VO_4_ (100 μM), NaF (5 mM), sodium pyrophosphate (10 mM), protease inhibitor cocktail] using stainless-steel beads and a Qiagen Tissue Lyser (30 Hz for 5 min). Homogenates were centrifuged at 4°C for 10 min at 14,000 *g*, after which the protein concentration of supernatants was determined by Bradford assay. Lysates of equal protein concentration were prepared in 2× Laemmli buffer containing 2-mercaptoethanol and heated at 37°C (for mitochondrial proteins) or 95°C (for all nonmitochondrial proteins) for 5 min. Proteins were separated by SDS-PAGE and transferred to nitrocellulose membranes for Western blotting. After ponceau staining to ensure equal protein loading, membranes were blocked in BSA for 1 h and incubated overnight in total OXPHOS rodent WB antibody cocktail (no. ab110413; Abcam), anti-PGC-1α (no. SAB4200209; Sigma), anti-phospho AMPK Thr^172^ (Cell Signaling Technology no. 2535S), anti-AMPK (no. 2793S; Cell Signaling Technology), anti-phospho S6K Thr^389^ (no. 9206; Cell Signaling Technology), anti-S6K (no. 2708; Cell Signaling Technology), anti-phospho Akt Ser^473^ (no. 4060; Cell Signaling Technology), anti-Akt (no. 9272; Cell Signaling Technology), anti-LC3 (no. 12741; Cell Signaling Technology), or anti-β-actin (no. 60008-1-Ig; Proteintech) at 4°C. Blots were visualized after a 1-h incubation with infrared anti-mouse or anti-rabbit secondary antibody, using a LI-COR Odyssey fluorescent Western blotting system. Protein expression was quantified using densitometry (Image Studio Lite; LI-COR) and normalized to Akt, which was unchanged by the treatments. LC3 was presented as the ratio of light chain 3 (LC3)II/LC3I as prescribed (25).

#### Citrate synthase activity.

Muscle homogenates were prepared in KCl-EDTA buffer (pH 7.4) from ∼10 to 40 mg of frozen quadriceps. Following three freeze-thaw cycles, samples were centrifuged at 4°C for 10 min at 1,000 *g* to settle cellular debris. Supernatants were analyzed for citrate synthase activity using a modified method (40). Briefly, aliquots of supernatant were added to the appropriate wells of a 96-well microplate containing an assay solution comprised of Tris (72.5 mM), acetyl-CoA (0.45 mM), and 5,5′-dithiobis-2-nitrobenzoate (DTNB; 0.1 mM) at a pH of 8.3. After the plate was monitored for possible background activity, activity reactions were initiated by the addition of oxaloacetic acid (0.5 mM) to each well. Changes in absorbance at 405 nm were recorded for each well every 9–11 s over 3 min at room temperature. Citrate synthase activity was calculated using the extinction coefficient for DTNB (which is reduced by the CoA-SH released during the cleavage of acetyl-CoA by citrate synthase).

#### Statistics.

All raw data, and analysis scripts are available at http://bridgeslab.github.io/ObesityParticulateTreatment (42). Statistics and calculations were performed using R version 3.1.1 (34). For longitudinal data, mixed linear models were used and χ^2^ tests performed to determine the significance of the MCP230 treatment. Mixed linear models used the R package lme4 (version 1.1–7; Ref. 4). In all cases, normality of the data and models were determined via Shapiro-Wilk Test, and equal variance was tested using Levene's test from the car package (version 2.0–21; Ref. 19). Pairwise Student's *t*-test, Welch's *t*-test, or Wilcoxon rank sum tests were performed as indicated in results and the figure legends and were dependent on normality and homoscedasticity. In cases where cabosil and saline treatment were not significantly different, these data were combined and designated as a single control group. For energy expenditure calculations, we performed an ANCOVA analysis with lean body mass and the treatment group as noninteracting covariates and the averaged light or dark V̇o_2_ as the responding variable as described previously (48). Statistical significance was designated as *P* < 0.05.

## RESULTS

### 

#### Gestational exposure to MCP230 leads to increased body size on a high-fat diet.

**Fig. 1.**
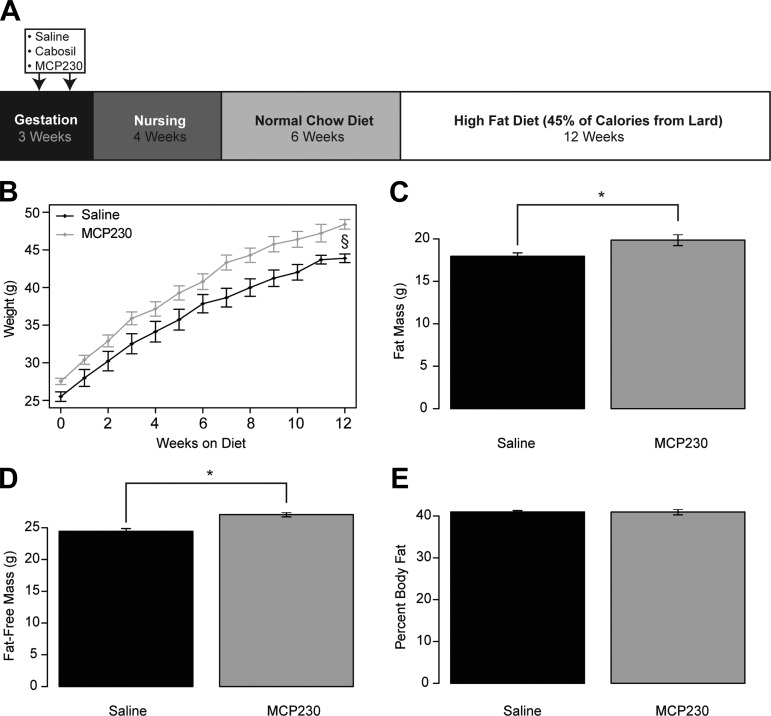
In utero exposure to laboratory-generated, combustion-derived particular matter (MCP230) results in increased body size. *A*: schematic of the experimental design. *B*: body weight throughout the high-fat diet phase of the intervention. *C*–*E*: absolute body fat (*C*), fat-free mass (*D*), and %body fat (*E*) after 12 wk of high-fat diet Zeitgerber time (ZT)12. Data shown are group means ± SE. §*P* < 0.05 via mixed linear model, compared by χ^2^ test (*B*); *P < 0.05 via Student's *t*-test (*C* and *D*). Black bars, saline-exposed mice; gray bars, MCP230-exposed mice.

To test the metabolic effects of gestational exposure to EPFRs, pregnant females were exposed to MCP230 on *days 10* and *17* of gestation. As controls, mice were exposed to either cabosil (the nonconjugated particulate without the EPFR group) or saline. After birth, these mice were left with their dams until weaning onto standard rodent chow at 28 days of age. At 10 wk of age, male mice were placed on a high-fat diet (HFD) consisting of 45% of calories from lard to induce obesity (Fig. 1*A*).

As shown in Fig. 1*B*, at 10 wk of age, mice that were exposed to MCP230 had a 7.6% higher body weight than the saline-exposed mice and remained heavier, gaining more weight throughout the HFD phase (*P* = 3.5 × 10^−5^). After 12 wk of HFD, the MCP230-exposed mice were 4.5 g heavier than saline-exposed mice (9.8%, *P* < 0.001; Fig. 1*B*). We assessed body composition after 12 wk of HFD and observed significant elevations in both fat mass (10.1% increase, *P* = 0.011) and fat-free mass (10.2% increase, *P* = 2.2 × 10^−4^) in the MCP230-exposed mice (Fig. 1, *C* and *D*). The relative adiposity of these mice, as determined by the percent fat mass, was not different between groups (Fig. 1*E*).

#### MCP230-exposed mice have reduced caloric intake and increased serum concentrations of leptin, ghrelin, and GLP-1.

**Fig. 2.**
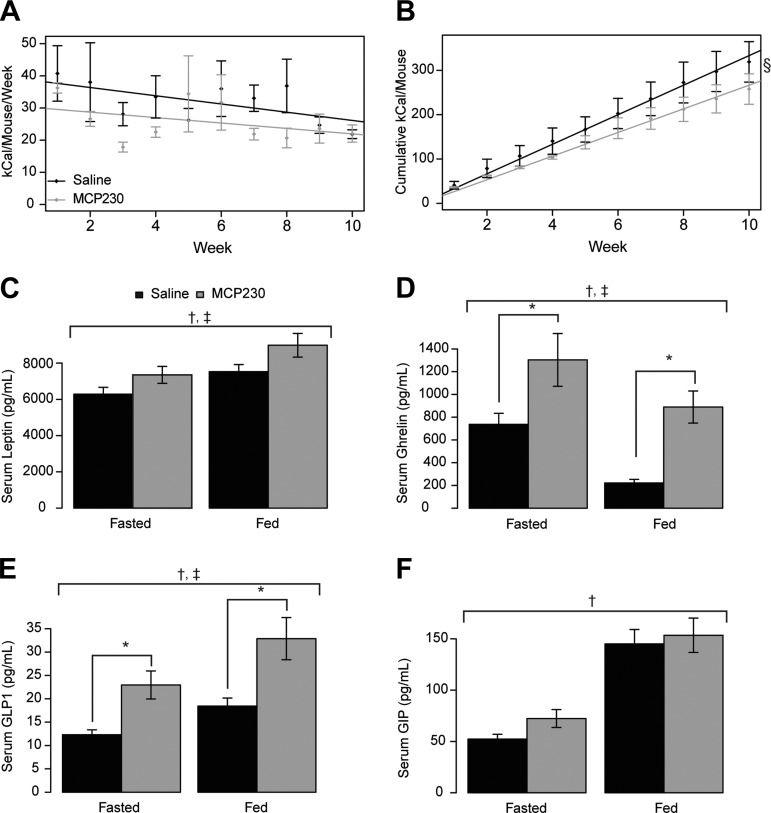
Gestational exposure to MCP230 causes a reduction in food intake and alters “hunger hormone” concentrations on a high-fat diet. *A* and *B*: food intake per mouse was calculated on a weekly (*A*) and cumulative (*B*) basis throughout the high-fat diet phase of the intervention. *C*–*E*: MCP230-exposed mice had elevated serum concentrations of leptin (*C*), ghrelin (*D*), and glucagon-like peptide-1 (GLP-1; *E*) after access to the high-fat diet. *F*: serum glucose-dependent insulinotropic polypeptide (GIP) tended to be elevated during the fasted state, although this did not attain statistical significance. Fed serum was collected at ZT12. Fasting serum was collected following an overnight fast (∼16 h) at ZT4. Data shown are group means ± SE; *n* = 8–14/group. §*P* < 0.05 by mixed linear model, compared by χ^2^ test (*B*); †main effect for feeding state (*C*–*F*); ‡main effect for MCP230 exposure by 2-way ANOVA (*C*–*E*); *P < 0.05 via a Wilcoxon rank sum test (*D* and *E*). Black bars, saline-exposed mice; gray bars, MCP230-exposed mice.

To determine how energy balance was affected in MCP230-exposed mice, we examined their food intake throughout the HFD feeding period. As shown in Fig. 2*A*, all mice tended to eat less food each week, although this did not reach statistical significance. Cumulatively, the MCP230-exposed mice ate less food throughout the diet (−6.3 ± 1.8 kcal·wk^−1^·mouse^−1^, *P* = 8.0 × 10^−4^; Fig. 2*B*). Throughout the 12-wk HFD treatment, this corresponds to a 19.2% reduction in total caloric intake. During the metabolic cage experiments, which occurred prior to HFD feeding, the MCP230-exposed mice tended to eat less food per feeding bout, whereas each feeding bout also tended to be shorter in duration; however, neither of these parameters were significantly different (data not shown). There were no differences between groups for the frequency of feeding bouts.

Leptin concentrations were modestly elevated in serum from MCP230-exposed mice (main effects feeding state, *P* = 0.002, and treatment, *P* = 0.011, by 2-way ANOVA, with post hoc *t-*test *P* values of 0.058 under fasting and *P* = 0.097 under fed conditions; Fig. 2*C*). Elevations in circulating leptin levels are consistent with the increase in fat mass observed in MCP230-exposed mice (Fig. 1*C*). We observed significant serum elevations in both the fasting and fed states for ghrelin (for main effects of feeding state *P* = 0.001, and MCP230 treatment *P* = 6.5 × 10^−6^, with post hoc *t-*test *P* values of 0.024 in the fasting and *P* = 0.0002 in the fed state; Fig. 2*D*), which is consistent with a reduction in food intake (Fig. 2, *A* and *B*) and reduced energy expenditure (Fig. 4, *A*–*E*) observed in the MCP230-exposed mice (11, 46, 47, 54). Similarly, GLP-1 was elevated in serum from MCP230-exposed mice in both the fasted and fed states (main effects for feeding state *P* = 0.002, and treatment *P* = 3.6 × 10^−5^, with post hoc *t-*test *P* values of 0.024 in the fasted and *P* = 0.001 in the fed state; Fig. 2*D*), which is also consistent with the MCP230-exposed mice eating less (Fig. 2, *A* and *B*) (3, 49). There was an effect of the feeding state with respect to glucose-dependent insulinotropic polypeptide (GIP) concentrations (*P* = 6.0 × 10^−9^ by 2-way ANOVA; Fig. 2*F*), and GIP was elevated in serum from MCP230-exposed mice in the fasted state, although these values did not quite attain statistical significance (*P* = 0.069 by Wilcoxon rank sum test). Although there were main effects of the feeding state for PAI-1 and resistin levels, these were not different between the two treatment groups (data not shown).

**Fig. 3.**
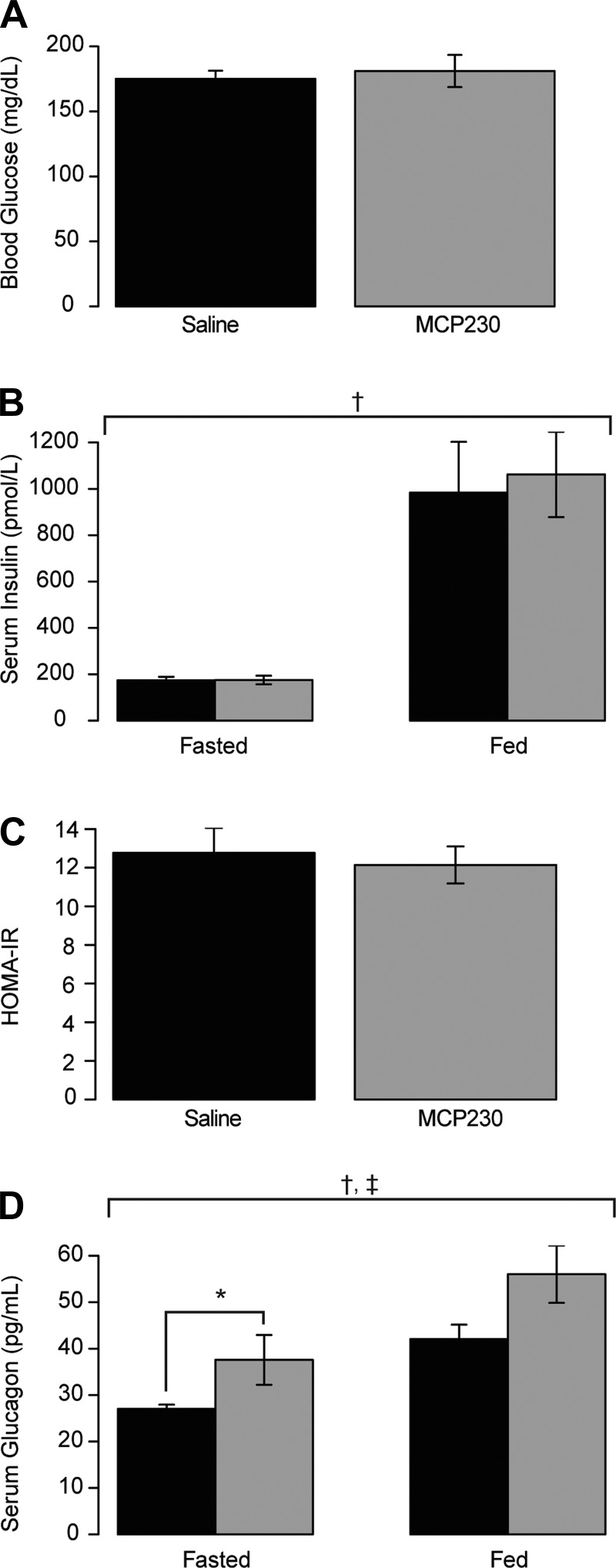
Gestational exposure to MCP230 causes an increase in serum glucagon but does not differentially alter glucose or insulin concentrations following exposure to a high-fat diet. Fasting blood glucose (*A*), serum insulin (*B*), homeostatic model assessment of insulin resistnace (HOMA-IR; *C*), and serum glucagon (*D*) concentrations were determined after a 16-h fast at ∼ZT4. Fed serum was collected at ZT12 and analyzed for insulin (*B*) and glucagon (*D*). Data shown are group means ± SE; *n* = 8–14/group. †Main effect for feeding state (*B* and *D*); ‡main effect for MCP230 exposure by 2-way ANOVA (*D*); *P < 0.05 via a Wilcoxon rank sum test (*D*). Black bars, saline-exposed mice; gray bars, MCP230-exposed mice.

We next evaluated the extent of obesity-related comorbidities in these mice after 12 wk of high-fat feeding. We observed no differences in fasting blood glucose as a result of MCP230 exposure (Fig. 3*A*). As shown in Fig. 3*B*, there was a main effect of feeding state on serum insulin concentrations (*P* = 3.3 × 10^−6^); however, MCP230 exposure had no effect. Calculation of the HOMA-IR revealed that both the saline and MCP230-exposed groups had similar HOMA-IR scores (12.77 ± 1.29 vs. 12.14 ± 0.96 for saline and MCP230, respectively, *P* = 0.74; Fig. 3*C*). Taken together, these data indicate that although HFD did impair insulin sensitivity, there was no difference between these two groups. Consistent with this, we observed no changes in the levels of fasted Akt phosphorylation in muscle tissue (data not shown). With respect to glucagon levels, both feeding state (*P* = 7.3 × 10^−5^) and MCP230 treatment (*P* = 4.0 × 10^−3^) increased serum glucagon concentrations. MCP230-exposed mice had elevated glucagon concentrations in the fasted and fed state, although fed state levels did not quite attain statistical significance (32.65%, *P* = 0.009 for fasting and 28.46%, *P* = 0.059 for fed by post hoc Wilcoxon rank sum tests; Fig. 3*D*).

#### MCP230 mice have reduced energy expenditure.

**Fig. 4.**
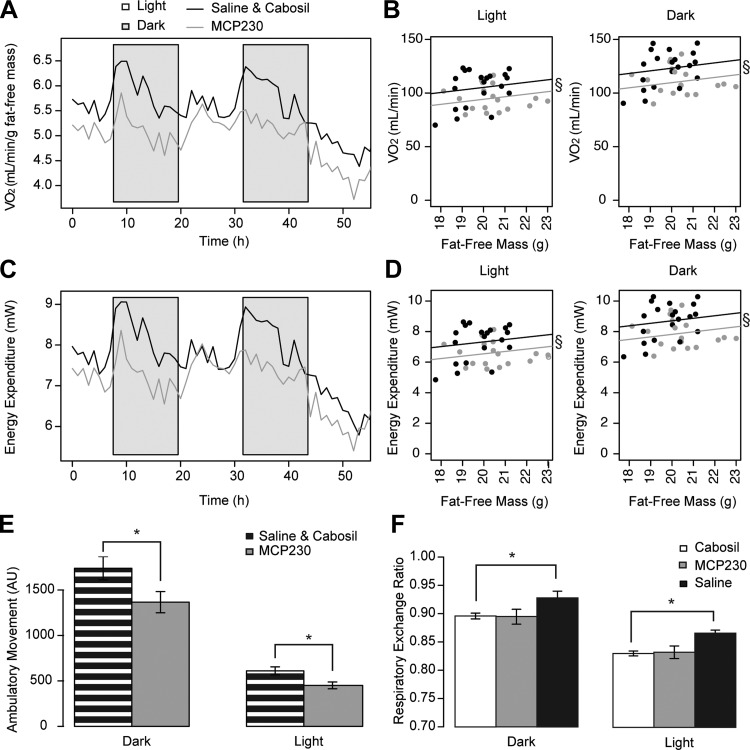
In utero exposure to MCP230 reduces energy expenditure and lowers physical activity. *A* and *B*: O_2_ consumption rates (V̇o_2_; *A*) and V̇o_2_ analysis (*B*) normalized to fat-free mass during both the light and dark phases. Each circle represents the average O_2_ consumption of each mouse. *C* and *D*: time course of energy expenditure (*C*) and energy expenditure normalized to fat-free mass (*D*) during both the light and dark phases. Each circle represents the average energy expenditure of each mouse. *E*: quantification of ambulatory movement during the light and dark phases. *F*: respiratory exchange ratio of each group. Saline and cabosil groups were not combined for this analysis because there was a significant reduction in the respiratory exchange ratio for both the cabosil- and MCP230-exposed groups. Data shown are either individual (*B* and *D*) or group means (*A*, *C*, *E*, and *F*) ± SE (*E* and *F*); *n* = 18, 6, or 14 for MCP230, saline, and cabosil groups, respectively. §*P* < 0.05 by ANCOVA (*B*); *P < 0.05 by Student's *t*-test (*E*) or Wilcoxon rank sum test (*F*). Black bars, saline-exposed mice; open bars, cabosil-exposed mice; gray bars, MCP230-exposed mice; black and white striped bars, combined saline- and cabosil-exposed groups.

Since the MCP230 mice did not appear to be larger due to excessive caloric intake, we next examined their energy utilization. To evaluate energy expenditure, we individually housed 9-wk-old mice (prior to HFD) in metabolic cages for indirect calorimetry, physical activity monitoring, and evaluation of gas exchange rates. As shown in Fig. 4, *A* and *B*, the MCP230-exposed mice had lower oxygen consumption (V̇o_2_) in both the light and dark phases (−19.1%, *P* = 0.031, and −16.8%, *P* = 0.019, respectively). This reduction in V̇o_2_ translated to a similar reduction in energy expenditure in both the light and dark phases (−18.4%, *P* = 0.032, and −16.4%, *P* = 0.021, respectively; Fig. 4, *C* and *D*). In Fig. 4, *B* and *D*, each circle represents the average value for each individual mouse plotted against fat-free mass. Accounting for change in lean mass is necessary due to known associations between this covariate and rates of oxygen consumption (48). To determine whether these decreases in energy expenditure were associated with changes in physical activity, we monitored the ambulatory movements of these mice while they were housed in the metabolic cages. As shown in Fig. 4*E*, compared with the control group, we observed 21.4 (*P* = 0.040) and 26.2% (*P* = 0.0099) reductions in physical activity for the MCP230-exposed mice in the dark and light phases, respectively.

Next, we evaluated energy substrate preference by analyzing the respiratory exchange ratio of the three groups. When this ratio nears 1, that indicates preference for predominately carbohydrate as fuel, and as it nears 0.7 it indicates utilization of mainly lipids (5). Although there was no difference in the respiratory exchange ratio between MCP230- and cabosil-exposed mice, we did observe a significant elevation (carbohydrate preference) in the saline-exposed mice during both the light and dark phases relative to mice exposed to either the vehicle control (cabosil) or MCP230 (the EPFR) (Fig. 4*F*). These data indicate that particle exposure alone (cabosil) altered substrate preference but that exposure to the EPFR did not alter substrate preference.

#### Skeletal muscle from MCP230-treated mice have reduced mtDNA copy number and a lower citrate synthase activity.

**Fig. 5.**
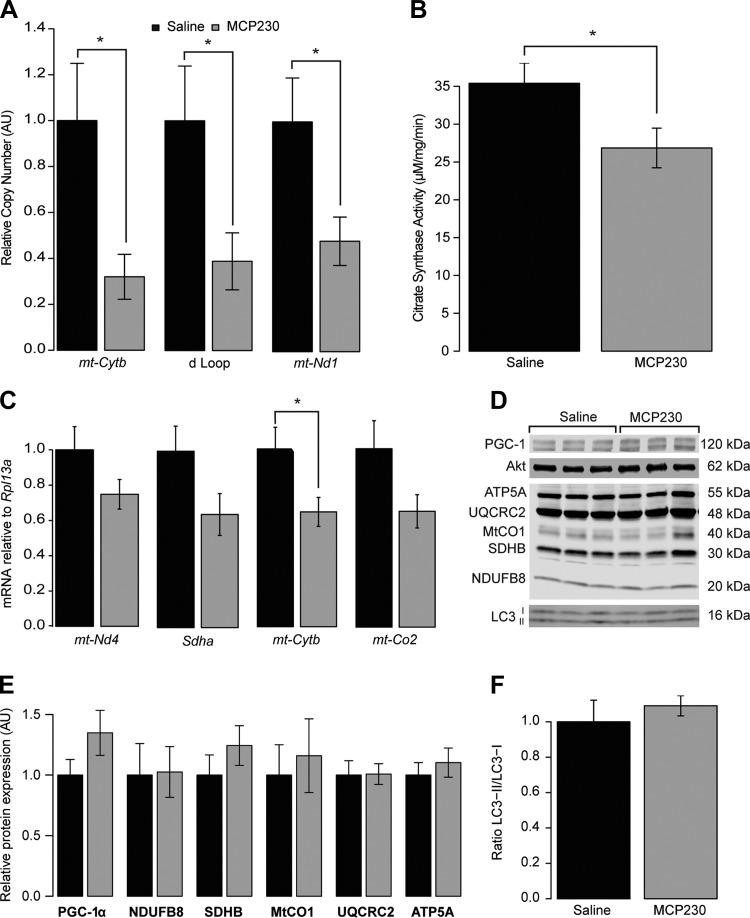
Exposure to MCP230 in utero results in skeletal muscle mitochondrial abnormalities following high-fat diet consumption as adults. mtDNA copy number (*A*), citrate synthase activity (*B*), and mRNA levels of oxidative phosphorylation genes (*C*) were reduced in the quadriceps muscles of mice that were indirectly exposed to MCP230 in utero and subjected to 12 wk of high-fat diet as adults. Quadriceps PPARγ coactivator-1 α (PGC-1α), light chain 3 (LC3), and select electron transport chain protein expression was unchanged in the MCP230-exposed mice (*D*, representative blots, and *E* and *F*, relative quantification). Data shown are group means ± SE. *P < 0.05 via Student's *t*-test; *n* = 7–12/group. Black bars, saline-exposed mice; gray bars, MCP230-exposed mice. ATP5A, ATP synthase H+ transporting α-subunit; UQCRC2, ubiquinol-cytochrome *c* reductase core protein II; MtCO1, mitochondrially encoded cytochrome *c* oxidase I; SDHB, succinate dehydrogenase complex, subunit B; NDUFB8, NADH, ubiquinone oxidoreductase subunit B8.

Because of the observed reductions in whole body oxygen consumption and total energy expenditure, we next explored the hypothesis that MCP230-exposed mice have skeletal muscle mitochondrial deficits, as muscle is the major organ responsible for variations in resting energy expenditure (57). To test this, we first determined mitochondrial DNA (mtDNA) copy number in quadriceps muscle after the 12-wk HFD phase. Figure 5*A* demonstrates that MCP230-exposed mice have a marked reduction in mtDNA copy number relative to the saline-exposed mice, as determined using primers designed for three distinct mtDNA-encoded gene regions. Decreases of 61.2, 68.0, and 51.9% were observed for the mitochondrial D-loop, *mt-Cytb*, and *mt-Nd1*, respectively (*P* = 0.039, *P* = 0.031, and *P* = 0.032, respectively), suggesting that MCP230-exposed mice may have reduced skeletal muscle mitochondrial content. Since citrate synthase activity is better associated with skeletal muscle mitochondrial content than mtDNA copy number (and is also a good indicator of tricarboxylic acid cycle activity; see Ref. 27), we measured citrate synthase activity to further evaluate mitochondrial content and function in the skeletal muscle from MCP230-exposed mice. As shown in Fig. 5*B*, maximal citrate synthase activity was reduced 24.1% in the quadriceps from MCP230-exposed mice (*P* = 0.03). Together, reduced mtDNA copy number and lower citrate synthase activity suggest that mice exposed to MCP230 may have reduced mitochondrial oxidative enzyme content and, as a result, reduced skeletal muscle oxidative capacity, which, along with the reduction in physical activity, would likely contribute to the reduced V̇o_2_ seen in these mice. Consistent with this hypothesis, mRNA transcript levels for the mitochondrial- and nuclear-encoded electron transport genes *mt-Nd4* (25.2%), *Sdha* (35.9%), *mt-Cytb* (35.4%), and *mt-Co2* (35.1%) were reduced in the quadriceps from MCP230-exposed mice, although not all of these reductions attained statistical significance (*P* = 0.12, *P* = 0.08, *P* = 0.04, and *P* = 0.10, respectively; see Fig. 5*C*). To determine whether differences in skeletal muscle mitochondrial electron transport enzymes were present at the protein level, we measured the relative expression of several electron transport chain proteins via Western blotting (Fig. 5*D*). We did not observe differences in levels of any of the five oxidative phosphorylation proteins measured in skeletal muscle, nor did we see changes in PGC-1α protein expression (Fig. 5, *D* and *E*) with MCP230 exposure. To test whether the lack of change in mitochondrial protein expression was due to suppression of autophagy, we blotted for processing of LC3 and observed no evidence of decreased autophagy (Fig. 5, *D* and *F*). We did not see alterations in any of the other measured markers of skeletal muscle metabolism and growth (phospho-Akt Ser^473^, phospho-AMPK Thr^172^, or phospho-S6K Thr^389^; data not shown).

**Fig. 6.**
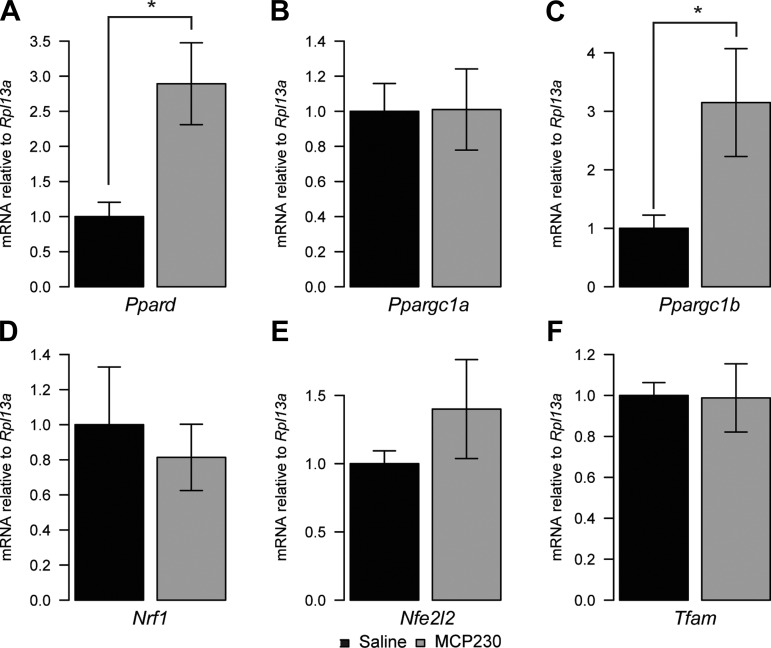
Indirect exposure to MCP230 in utero is not associated with reductions in the mRNA of upstream regulators of mitochondrial biogenesis *Ppard* (*A*) and *Ppargc1b* (*C*). mRNA was elevated in the MCP230-exposed mice, whereas *Ppargc1a* (*B*), *Nrf1* (*D*), *Nfe2l2* (*E*), and *Tfam* mRNA (*F*) were not different. Data shown are group means ± SE. *P < 0.05 via Student's *t*-test (*A*) or Wilcoxon rank sum test (*C*); *n* = 7–12/group. Black bars, saline-exposed mice; gray bars, MCP230-exposed mice.

**Fig. 7.**
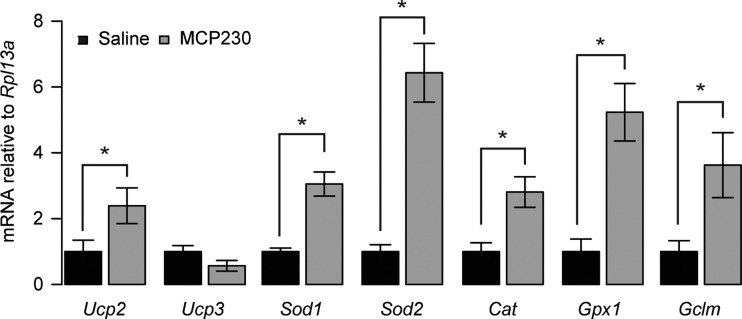
The antioxidant defense system is upregulated in the quadriceps of MCP230-exposed mice. Data shown are group means ± SE. *P < 0.05 via Student's *t*-test (*Cat*), Welch's *t*-test (*Sod1*, *Sod2*, *Gpx1*), or Wilcoxon rank sum test (*Ucp2*, *Gclm*); *n* = 7–12/group. Black bars, saline-exposed mice; gray bars, MCP230-exposed mice.

To test whether reductions in mtDNA copy number and citrate synthase activity were due to lowered mitochondrial biogenesis, we evaluated the expression level of several known mitochondrial biogenesis genes. Although we observed increases in the mRNA of *Ppard* and *Ppargc1b* (Fig. 6, *A* and *C*), there were no differences in the expression levels of *Ppargc1a*, *Nrf1*, *Nfe2l2*, or *Tfam* (Fig. 6, *B* and *D*–*F*). In the absence of reduced mitochondrial biogenesis markers or changes in the levels of oxidative phosoporylation enzymes, the mitochondrial deficits we observe in the skeletal muscle of mice exposed to MCP230 may be a response to oxidative stress rather than transcriptional downregulation of mitochondrial biogenesis per se. In support of this notion, we found robust increases in the mRNA for *Ucp2*, *Sod1*, *Sod2*, *Cat*, *Gpx1*, and *Gclm*, enzymes activated in response to oxidative stress (Fig. 7).

## DISCUSSION

Although epidemiological studies have linked exposure to particulate matter (PM) with obesity and its comorbidities, few have evaluated energy expenditure changes in response to acute gestational exposure. In this study, we tested some of the metabolic effects of a limited gestational exposure to a recently realized environmental pollutant that is present in most combustion-derived PM: EPFRs. Each exposure of MCP230 that the mothers received was the equivalent to a human breathing 200 μg/m^3^ of EPFR, which is similar to what would be inhaled on a typical day in one of the major US cities (38). We noted that pups born from mothers that were acutely exposed to PM grew larger, despite reductions in food intake, and that this was associated with reduced energy expenditure and mitochondrial impairments in skeletal muscle.

One potential explanation for reductions in energy expenditure and skeletal muscle mitochondrial function is the observed reduction in physical activity for MCP230-exposed mice. It is also possible that muscle weakness (due to reduced skeletal muscle oxidative capacity; see Refs. 44 and 57) could contribute to the reduced physical activity of MCP230-exposed animals. Both of these hypotheses are consistent with cross-sectional studies showing negative associations between ambient air pollutant exposure and leisure time physical activity (35) and exercise performance (14, 33, 36). Our current data are unable to distinguish whether reduced mitochondrial function is the primary cause of these reductions in energy expenditure or whether this observation is secondary to a reduced propensity for physical activity or some other mechanism. However, our observations of reductions in mtDNA, citrate synthase activity, and mRNA transcripts support the hypothesis that gestational exposure to EPFRs can affect skeletal muscle mitochondrial oxidative function, which would contribute to the overall changes we observe in energy expenditure.

The mechanisms by which gestational exposure to EPFRs result in reduced mitochondrial function are not yet clear. Our data are consistent with chronic models of PM_2.5_ exposure, which show reduced mitochondrial numbers in white adipose tissue (55, 56). Analyses of placental tissues from mothers showed a strong correlation between late-gestational PM_10_ exposure and placental mtDNA content (22). Given the elevated sensitivity of mitochondria to free radicals and oxidative stress, it is reasonable to hypothesize that, during development, EPFR-mediated mitochondrial damage may result in chronic decreases in mitochondrial oxidative function either directly via reactive oxygen species (ROS) or indirectly via inflammatory processes. Indeed, the bioenergetics proteins in skeletal muscle are highly susceptible to ROS-induced posttranslational modifications, changes thought to be important for reducing endogenous ROS production and protect against irreversible oxidative damage during periods of cellular stress (26). In line with this concept, Siegel et al. (39) have shown that mild oxidative stress in vivo impairs skeletal muscle oxidative efficiency and reduces oxidative phosphorylation coupling without altering the expression of key electron transport chain proteins or their respiratory activities ex vivo. This suggests that reduced skeletal muscle oxidative capacity in response to oxidative stress is probably not due to downregulation of the mitochondrial biogenesis pathways or irreversible oxidative damage to bioenergetic proteins. Similarly to previous reports on oxidative stress-induced mitochondrial dysfunction (39), we did not observe decreases in upstream regulators of mitochondrial biogenesis, increases in autophagy, or changes in mitochondrial protein expression as part of the chronic effects of acute in utero MCP230 exposure. However, we did observe marked increases in the transcripts of key enzymes of the antioxidant defense system (*Sod1*, *Sod2*, *Cat*, and *Gpx1*) as well as increased expression of *Ucp2*, an uncoupling protein known to be upregulated as a means to reduce endogenous ROS production (Fig. 7) (1, 17), and increases in both the nuclear receptor *Ppard* and the transcriptional coregulator *Ppargc1β*, both of which are required for the induction of *Sod1* and *Sod2* (20, 50). Based on our current protocol, mice are exposed to EPFRs after inheritance of maternal mitochondria, indicating that this mitochondrial damage occurs in situ in the progeny. Importantly, we have also previously observed signs of oxidative stress in the pregnant dams exposed to MCP230 (52). It should be emphasized that in our case, EPFR exposure is indirect through the mother, as there is no evidence at present that the conjugated EPFR crosses the placenta to exert its effect on the muscle directly. However, we hypothesize that the changes we observe in the skeletal muscle mitochondria of the MCP230-exposed mice are at least in part a consequence of ROS-induced posttranslational changes and chronic oxidative stress. Future studies with more direct measurements of mitochondrial function and the oxidative stress response will provide more mechanistic insight into this process.

In contrast to previous studies that use chronic pollution models (1, 5, 9, 33, 35), we did not observe any indications that glycemic control was impaired to a greater extent in MCP230-exposed mice compared with the control groups following the HFD despite differences in fat mass. It should be noted that all of the mice in this study received the HFD to induce obesity and its metabolic comorbidities, and although we did not measure fasting glucose or insulin concentrations prior to the change in diet, the fasting glucose and insulin concentrations of all mice post-HFD were elevated compared with chow-fed mice of a similar age, regardless of exposures. There were no differences in fasting glucose, insulin, HOMA-IR score (Fig. 3, *A*–*C*), or Akt phosphorylation in muscle tissue (data not shown). We did not measure insulin sensitivity directly, which limits our ability to make strong conclusions about the effects of acute in utero PM exposure on insulin sensitivity. That said, our data suggest that the effects of acute gestational particulate exposure may not mimic the effects of chronic exposure, and the risk profiles and mechanisms associated with these exposures may differ.

In conclusion, we have investigated the effects of limited gestational exposure to combustion-derived pollutants in a mouse model of diet-induced obesity. Our findings show that even brief gestational exposure to environmental pollutants such as EPFRs can result in chronic changes in growth, metabolism, and energy balance. These changes are associated with skeletal muscle mitochondrial deficits and reductions in physical activity, which likely contribute to reduced energy expenditure and a predisposition to elevated body weight when exposed to a HFD. Although the mechanisms behind these changes remain to be determined, the finding that limited in utero exposure to EPFRs can affect energy metabolism later in life highlights a need for further research in this area.

## GRANTS

We acknowledge funding from the Memphis Research Consortium (to J. C. Han and D. Bridges), National Institute of Diabetes and Digestive and Kidney Diseases Grant R01-DK-107535, Le Bonheur Grant no. 650700 (to D. Bridges), and National Institutes of Health Grants (R01-AI-090059, R01-ES-015050, and P42ES013648) to S. A. Cormier.

## DISCLOSURES

The authors have no conflicts of interest, financial or otherwise, to disclose.

## AUTHOR CONTRIBUTIONS

E.J.S., A.R., S.J., J.P., M.J.P., and D.B. performed experiments; E.J.S., A.R., S.J., J.S., S.A.C., and D.B. analyzed data; E.J.S., A.R., J.C.H., S.A.C., and D.B. interpreted results of experiments; E.J.S. prepared figures; E.J.S. and S.A.C. drafted manuscript; E.J.S., A.R., S.J., J.C.H., S.A.C., and D.B. edited and revised manuscript; E.J.S., A.R., S.J., J.R.R., J.P., M.J.P., J.S., J.C.H., S.A.C., and D.B. approved final version of manuscript; S.A.C. and D.B. conception and design of research.
